# Association of Physical Activity from Wearable Devices and Chronic Disease Risk: Insights from the All of Us Research Program

**DOI:** 10.21203/rs.3.rs-6263507/v1

**Published:** 2025-05-07

**Authors:** Rui Zhang, Yu Hou, Erjia Cui, Kelvin Lim, Lisa Chow, Michael Howell, Sayeed Ikramuddin

**Affiliations:** University of Minnesota; University of Minnesota; University of Minnesota, Division of Biostatistics; University of Minnesota; University of Minnesota; University of Minnesota; University of Minnesota

## Abstract

Physical activity is a modifiable factor influencing chronic disease risk. Previous studies often relied on self-reported activity measures or short-term assessments, limiting their accuracy. Leveraging Fitbit-derived data from the All of Us Research Program, we investigated associations between long-term physical activity patterns and chronic disease incidence in a diverse cohort. The study included 22,019 participants with at least six months of Fitbit monitoring and linked electronic health records. Key activity metrics included daily step count, activity calories, elevation gain, and activity duration at different intensities. Higher physical activity levels were associated with a lower risk of multiple chronic diseases. A 2,000-step increase in daily step count was linked to a reduced risk of obesity (hazard ratio [HR] = 0.85, 95% confidence interval [CI]: 0.80–0.90), type 2 diabetes (HR = 0.78, CI: 0.72–0.84), and major depressive disorder (HR = 0.83, CI: 0.77–0.90). Elevation gain was inversely associated with obesity (HR = 0.86, CI: 0.78–0.95) and type 2 diabetes (HR = 0.65, CI: 0.53–0.80). Increased time spent in very active intensity correlated with a lower risk of multiple conditions, including obstructive sleep apnea and morbid obesity. Conversely, prolonged sedentary time was associated with an increased risk of cardiometabolic diseases, including obesity (HR = 1.08, CI: 1.06–1.10) and essential hypertension (HR = 1.05, CI: 1.04–1.07). A sensitivity analysis using BMI-defined obesity instead of EHR-based diagnoses confirmed the robustness of these associations. These findings underscore the protective role of increased physical activity and reduced sedentary time in mitigating chronic disease risk. They support the development of personalized physical activity recommendations and targeted public health interventions aimed at improving long-term health outcomes. Future research integrating machine learning approaches could further refine activity-based disease prevention strategies.

## Introduction

Chronic diseases, including cardiometabolic disorders, obesity, and major depressive disorder (MDD), are the main causes of morbidity and mortality worldwide^1–3^. Given the critical role of lifestyle factors in their onset and progression, physical activity has garnered increasing attention as a key modifiable determinant^4,5^. Wearable devices enable continuous, non-invasive monitoring of physical activity, offering novel opportunities to explore its association with chronic disease outcomes. Prior research has established links between physical activity and multiple chronic diseases, such as cardiovascular disease, type 2 diabetes, depression, Parkinson’s disease, and multiple sclerosis^4–8^. However, many studies rely on self-reported activity data or short-term objective assessments via accelerometers or pedometers. Self-reported data, though informative, often diverge from objectively measured activity levels^9,10^.

The emergence of commercial wearables, such as Fitbit, offers a means to address these limitations by enabling long-term, objective activity tracking. Fitbit-based studies have validated their accuracy in measuring steps, heart rate, and energy expenditure^11,12^. These devices generate longitudinal data, providing novel insights into individual activity patterns and facilitating large-scale epidemiological investigations of activity-related chronic disease risk.

The *All of Us* Research Program (AoU), a National Institutes of Health initiative, seeks to collect health data from over one million individuals across diverse U.S. populations^13,14^. The program integrates electronic health records (EHRs), genomic data, physical measurements, and participant surveys. As of Version 8, more than 59,000 participants have contributed Fitbit data, encompassing activity and sleep metrics. This dataset uniquely enables the first large-scale exploration of longitudinal associations between objectively measured activity behaviors and chronic disease outcomes in a diverse U.S. cohort.

This study leverages extensive Fitbit activity data and longitudinal EHRs from AoU to examine associations between real-world activity patterns and chronic disease incidence. We aim to elucidate how activity intensity, duration, and regularity influence chronic disease risk, with findings informing evidence-based lifestyle recommendations for disease prevention in the general U.S. population.

## Results

The AoU Research Program included 58,785 participants with available Fitbit activity data. Among them, 22,019 participants had both corresponding EHR data and at least six months of Fitbit monitoring (supplementary Figure. 1). The median participants age was 52 years (interquartile range: 38 to 63 years). The majority of participants were female (71.4%), followed by males (28.2%), with 0.4% having unknown sex information. Race distribution was as follows: White (77.5%), Black (6.3%), Asian (3.4%), Other (7.3%), and Unknown (5.5%). Hispanic or Latino participants comprised 7.8%, while 90.4% were non-Hispanic, and 1.9% had unknown ethnicity. The median Fitbit monitoring duration was 2.53 years (1.42, 4.32). Participants had a median daily step count of 6,166 steps (3,698, 8,600). Participants had a median daily step count of 6,166 steps (484.3, 1050.2), with a median basal metabolic rate (BMR) of 1,474.0 kcal calories (1,287.9, 1,686.9). The median total daily calories burned was 2,101.6 kcal (1,787.4, 2,507.4). Median daily elevation gain was 33.9 meters (4.3, 91.0). Daily median activity intensity metrics included sedentary time (738 min; 12.1–931.3), lightly active time (177.0 min; 118.3–227.5), fairly active time (9.6 min; 2.6–19.0), and very active time (13.0 min; 6.0–24.7) (Table 1).

### Associations between physical activity patterns and chronic disease incidence

**Figure 1:**
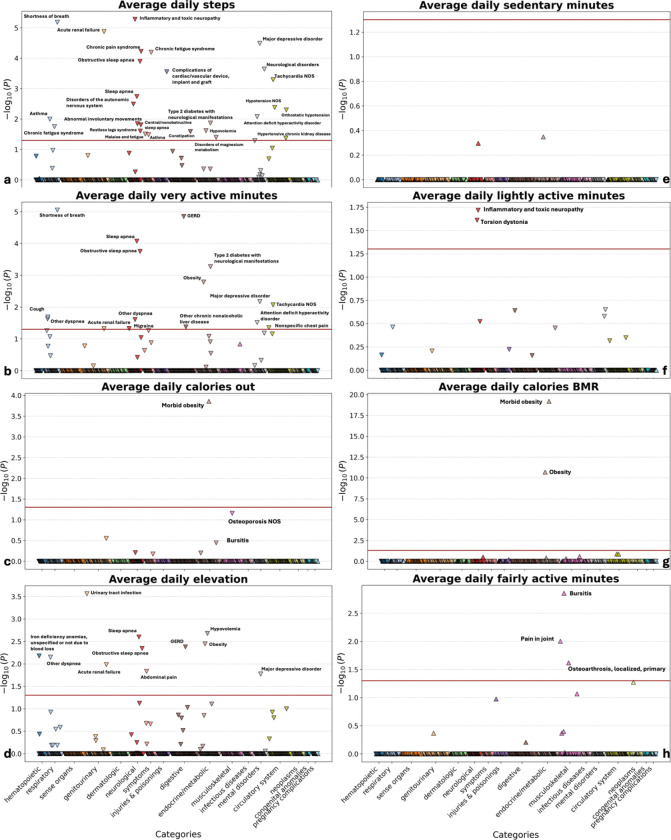
Associations between physical activity patterns and chronic disease incidence. (a). Major depressive disorder, obstructive sleep apnea, etc., are associated with daily steps. (b). Type II diabetes, obesity, etc., are associated with very active minutes. (c). Morbid obesity is associated with calories out. (d). Obesity, obstructive sleep apnea, etc., are associated with elevation. (f). Torsion dystonia, etc., are associated with lightly active minutes. (g). Morbid obesity and obesity are associated with calories BMR. (h). Pain in joint, etc., are associated with fairly active minutes.

After Bonferroni correction, 43 significant associations were identified between physical activity metrics and health outcomes. Daily step count was significantly associated with 27 diseases, including major depressive disorder and obstructive sleep apnea, while activity calories correlated with seven diseases, including sleep apnea and chronic fatigue syndrome. Morbid obesity and obesity were significantly associated with BMR-calories burned, with additional associations observed for total daily energy expenditure. Elevation gain was significantly linked to 11 diseases, including obesity and obstructive sleep apnea. Lightly active minutes were significantly associated with torsion dystonia and in ammatory neuropathy, fairly active minutes with three diseases (including joint pain), and very active minutes with 16 diseases (including type 2 diabetes and obesity) (Fig. 1).

**Figure 2:**
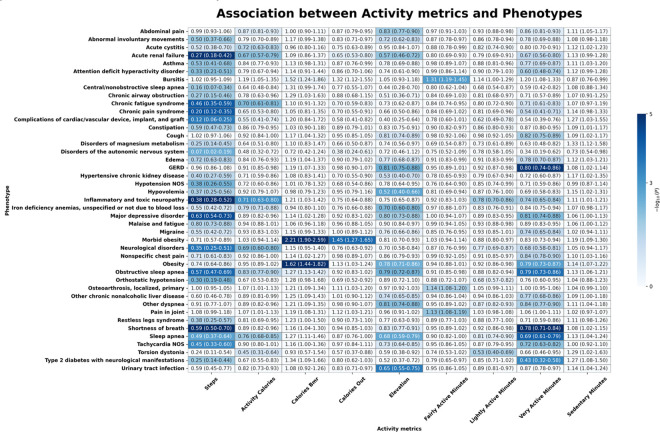
Heatmap of significant phenotypes across the physical activity patterns.

Figure 2 illustrates the associations between physical activity metrics and chronic disease incidence. An increase in daily step count was significantly associated with a lower risk of type 2 diabetes with neurological manifestations (odds ratio (OR) = 0.25; 95% confidence interval (CI) = 0.14–0.44; p-value = 0.01), major depressive disorder (OR = 0.63; 0.54–0.73; p < 0.01), sleep apnea (OR = 0.49; 0.37–0.64; p < 0.01), chronic fatigue syndrome (OR = 0.46; 0.35–0.59; p < 0.01), chronic pain syndrome (OR = 0.20; 0.12–0.35; p < 0.01), and obstructive sleep apnea (OSA) (OR = 0.57; 0.47–0.69; p < 0.01). Neurological disorders (OR = 0.35; 0.25–0.51; p < 0.01) and shortness of breath (OR = 0.49; 0.37–0.64; p < 0.01) were inversely associated with step count. Increased step count was also significantly associated with a lower risk of tachycardia NOS (OR = 0.45; 0.33–0.60; p < 0.01) and acute renal failure (OR = 0.27; 0.18–0.42; p < 0.01).

Higher BMR calories were significantly associated with an increased risk of obesity (OR = 1.62; 1.44–1.82; p < 0.01) and morbid obesity (OR = 2.22; 1.90–2.59; p < 0.01). Higher activity calories were associated with a reduced risk of sleep apnea (OR = 0.76; 0.68–0.85; p = 0.02), OSA (OR = 0.83; 0.77–0.90; p = 0.03), and chronic fatigue syndrome (OR = 0.70; 0.61–0.81; p < 0.01).

Greater daily elevation gain was significantly associated with a reduced risk of multiple conditions. Higher elevation gain was linked to lower risk of abdominal pain (OR = 0.83; 0.77–0.90; p = 0.01), urinary tract infection (OR = 0.65; 0.55–0.75; p < 0.01), gastroesophageal reflux disease (GERD) (OR = 0.81; 0.75–0.88; p < 0.01), and OSA (OR = 0.79; 0.72–0.87; p < 0.01). Elevation gain was also inversely associated with obesity risk (OR = 0.78; 0.71–0.86; p < 0.01). Major depressive disorder was also significantly associated with increased elevation gain (OR = 0.80; 0.73–0.88; p = 0.02), as were sleep apnea (OR = 0.68; 0.59–0.79; p < 0.01). Other conditions significantly associated with increased elevation gain included other dyspnea (OR = 0.81; 0.74–0.88; p < 0.01), and hypovolemia (OR = 0.52; 0.40–0.66; p < 0.01).

Greater time spent in very active minutes was significantly associated with a lower risk of OSA (OR = 0.79; 0.73–0.86; p < 0.01), obesity (OR = 0.79; 0.73–0.87; p < 0.01), and dyspnea (OR = 0.84; 0.77–0.90; p = 0.02). Fairly active minutes were significantly associated with an increased risk of bursitis (OR = 1.31; 1.19–1.45; p < 0.01), joint pain (OR = 1.13; 1.08–1.19; p < 0.01), and osteoarthrosis (OR = 1.14; 1.08–1.20; p = 0.02). Lightly active minutes were inversely associated with torsion dystonia risk (OR = 0.53; 0.40–0.69; p = 0.02).

### Cox Model Results for Physical Activity and Chronic Diseases

Cox proportional hazards regression models identified significant associations between physical activity metrics and chronic disease risk (Table 2, Supplementary Figures. 2–9). A 2,000-step increase in daily steps was associated with a reduced risk of obesity (HR = 0.85; 95% CI: 0.80–0.90), morbid obesity (HR = 0.79; 0.73–0.86), and type 2 diabetes (HR = 0.78; 0.72–0.84). Higher step counts were also associated with a reduced risk of major depressive disorder (HR = 0.83; 0.77–0.90). The risk of OSA decreased with increased step count (HR = 0.84; 0.79–0.89). Other dyspnea (HR = 0.84; 0.80–0.89) and essential hypertension (HR = 0.88; 0.84–0.92) also demonstrated a reduced risk with higher step counts.

Each 100-meter increase in elevation gain was associated with a lower risk of obesity (HR = 0.86; 0.78–0.95), morbid obesity (HR = 0.79; 0.67–0.92), type 2 diabetes (HR = 0.65; 0.53–0.80), and major depressive disorder (HR = 0.82; 0.71–0.95). OSA (HR = 0.83; 0.75–0.92), other dyspnea (HR = 0.87; 0.79–0.95), and essential hypertension (HR = 0.92; 0.86–0.99) were also inversely associated with elevation gain.

Greater sedentary time was positively associated with an increased risk of multiple conditions. Each additional hour of sedentary time was associated with a higher risk of obesity (HR = 1.08; 1.06–1.10), morbid obesity (HR = 1.05; 1.03–1.08), type 2 diabetes (HR = 1.04; 1.01–1.07), and OSA (HR = 1.03; 1.01–1.06). Other dyspnea (HR = 1.04; 1.02–1.06) and essential hypertension (HR = 1.05; 1.04–1.07) also exhibited increased risk with prolonged sedentary time.

Greater time spent in light activity was associated with a lower risk of several conditions. Each additional hour of light activity was associated with reduced risk of obesity (HR = 0.85; 0.79–0.91), morbid obesity (HR = 0.87; 0.79–0.95), type 2 diabetes (HR = 0.82; 0.74–0.91), major depressive disorder (HR = 0.84; 0.77–0.92), OSA (HR = 0.87; 0.81–0.94), other dyspnea (HR = 0.91; 0.85–0.97), essential hypertension (HR = 0.88; 0.83–0.93), and chronic pain syndrome (HR = 0.78; 0.64–0.95). Each additional hour of very active time was associated with a significantly lower risk of morbid obesity (HR = 0.45; 0.24–0.83), type 2 diabetes (HR = 0.34; 0.20–0.64), OSA (HR = 0.47; 0.32–0.70), other dyspnea (HR = 0.32; 0.22–0.47), essential hypertension (HR = 0.67; 0.51–0.88), and major depressive disorder (HR = 0.19; 0.44–0.77).

### Sensitivity Analysis using body mass index (BMI) for Obesity classification

To validate our findings, we performed a sensitivity analysis using BMI-defined obesity as an alternative outcome measure Logistic regression models assessing associations between physical activity metrics and BMI-defined obesity confirmed the primary findings. Higher daily step counts were associated with a significantly lower risk of obesity (OR = 0.79; 95% CI: 0.71–0.88; p < 0.01), as were increased elevation gain (OR = 0.77; 0.69–0.86; p < 0.01) and time spent in very active intensity (OR = 0.80; 0.72–0.88; p < 0.01). Conversely, BMR calories were positively associated with an increased risk of obesity (OR = 2.17; 1.82–2.60; p < 0.01), suggesting a potential compensatory metabolic effect. Cox proportional hazards models further corroborated these findings, demonstrating consistent associations between physical activity metrics and the risk of developing BMI-defined obesity over time (Supplementary Figure. 10). Higher daily step counts, increased elevation gain, and greater time spent in very active intensity were significantly associated with a lower hazard of obesity onset. These results underscore the robustness of our primary analysis and emphasize the protective role of increased physical activity against obesity, regardless of diagnostic criteria.

## Discussion

This study examined the associations between Fitbit-derived physical activity metrics and chronic disease incidence using data from the AoU Research Program. We utilized the latest AoU Curated Data Repository (CDR) version 8, released in February 2025, incorporating participant data up to October 1, 2023. With over 59,000 participants contributing Fitbit data, this dataset uniquely enables the exploration of long-term associations between objectively measured physical activity and chronic disease risk in a diverse cohort^15^. Utilizing this comprehensive dataset enhances the robustness and generalizability of our findings, ensuring their relevance to public health efforts and evidence-based physical activity interventions for disease prevention. Our findings suggest that higher physical activity levels—measured by daily step count, elevation gain, and activity intensity—are associated with a reduced risk of chronic diseases, including obesity, type 2 diabetes, major depressive disorder, and OSA. Conversely, prolonged sedentary time was linked to an increased risk of these conditions. These findings align with growing evidence emphasizing the role of regular physical activity in improving health and mitigating disease risk.

The inverse association between daily step count and the risk of obesity, type 2 diabetes, and major depressive disorder is consistent with prior research demonstrating the protective effects of increased step counts on cardiometabolic health and mental well-being^5,16–18^. Our results indicate that higher step counts are significantly associated with a lower risk of these conditions. This aligns with the concept that increased physical activity enhances energy expenditure, improves metabolic function, and alleviates depressive symptoms^4,19^. These findings reinforce public health recommendations advocating higher daily step counts as a practical strategy for chronic disease prevention.

Our study identified elevation gain, a less frequently examined metric, as significantly associated with a lower risk of several chronic diseases, including type 2 diabetes, morbid obesity, and OSA. Prior research suggests that elevation-related activities, such as stair climbing, may confer health benefits, though further evidence is needed to confirm their effects on cardiovascular and metabolic health^18,20^. These findings imply that integrating elevation-related activities into daily routines may offer additional health benefits. Further studies are warranted to elucidate the role of elevation-related activities in chronic disease prevention.

This study further highlighted the association between activity intensity and health outcomes. Greater time in very active intensity was significantly associated with a lower risk of obesity, type 2 diabetes, and OSA, while increased fairly active and lightly active time correlated with reduced risks of several chronic conditions, including major depressive disorder. These findings align with prior research demonstrating the benefits of varying physical activity intensities on cardiometabolic and mental health outcomes^21,22^. However, fairly active intensity was also associated with an increased risk of bursitis, joint pain, and osteoarthrosis, suggesting that while high-intensity activity is beneficial for chronic disease prevention, it may increase musculoskeletal strain. These findings underscore the need for personalized physical activity recommendations, particularly for individuals at risk of musculoskeletal injuries^23^.

Conversely, prolonged sedentary time was consistently associated with a higher risk of multiple chronic diseases, including obesity, type 2 diabetes, and essential hypertension. These findings align with previous studies highlighting the adverse effects of prolonged sedentary behavior on metabolic health and its association with an increased risk of cardiometabolic diseases^24–26^. The strong association between sedentary behavior and chronic disease risk underscores the importance of reducing sedentary time as a complementary strategy to increasing physical activity for improving health outcomes.

To evaluate the robustness of our findings, we performed a sensitivity analysis using BMI-defined obesity as an alternative outcome measure. Logistic regression analyses corroborated our primary findings, showing that higher daily step counts, increased elevation gain, and greater time spent in very active intensity were significantly associated with a lower risk of obesity. Cox proportional hazards models further reinforced these associations, demonstrating that physical activity metrics were consistently linked to a lower hazard of obesity development over time. These findings enhance the validity of our primary results, indicating that the observed associations persist regardless of whether obesity is defined by BMI criteria or clinical diagnosis. The sensitivity analysis underscores the robustness of the association between physical activity and obesity risk, suggesting that increasing daily movement and engaging in higher-intensity activities may confer benefits across varying obesity definitions.

A key strength of this study is the use of real-world wearable data, offering objective and granular insights into long-term physical activity patterns. Unlike traditional epidemiological studies relying on self-reported physical activity, wearable-derived metrics provide greater accuracy and allow for detailed assessments of activity intensity, duration, and elevation. However, several limitations should be considered. First, as an observational study, causality cannot be established. Although we mitigated reverse causation by excluding participants diagnosed within the initial 180 days, residual confounding remains possible. Furthermore, reliance on Fitbit users may introduce selection bias, as these individuals tend to be more health-conscious than the general population.

In conclusion, our findings highlight the importance of increasing daily steps, incorporating elevation-related activities, and engaging in diverse physical activity intensities to lower chronic disease risk.Equally important is reducing sedentary behavior to mitigate chronic disease risk. These findings offer valuable insights for designing targeted public health interventions that promote physical activity and reduce sedentary behavior, ultimately enhancing population health outcomes. Future research should investigate the underlying mechanisms of these associations and develop personalized activity recommendations to optimize health benefits while minimizing risks. With the growing application of machine learning in precision medicine^27–32^, integrating these approaches into future research could enhance the development of tailored physical activity interventions, enabling more precise health outcome predictions and personalized recommendations.

## Methods

### Study Participants

All participants provided informed consent to join the *All of Us* (AoU) Research Program, a large-scale National Institutes of Health (NIH)-funded initiative designed to advance research in health and disease^13,14^. Eligible participants included all U.S. adults (aged ≥ 18 years), excluding individuals who were incarcerated or unable to provide independent consent. This study utilized controlled-tier data (C2024Q3R4) from the AoU Researcher Workbench. At the time of analysis, data were available from 633,540 individuals enrolled between May 2018 and October 1, 2023^15^. Data collection included demographic information, survey responses from digital enrollment, physical measurements and vital signs recorded by partner healthcare provider organizations, voluntarily shared electronic health records (EHRs), and Fitbit data linked via participants’ Google Fitbit accounts within the AoU portal^13,14,33^.

### Fitbit activity data

This study utilized Fitbit-derived daily activity data to capture detailed physical activity patterns of participants. The dataset included activity calories, basal metabolic rate (BMR) calories, total calories burned, elevation gain, activity minutes at different intensity levels, and total step count. Fitbit data provides an advantage over traditional self-reported activity measures by offering objective, continuous, and non-invasive tracking. The inclusion of detailed activity metrics, such as light, moderate, and vigorous intensity levels, enables a comprehensive assessment of participants’ activity profiles over time. Fitbit data were collected from participants who linked their Fitbit accounts through the AoU portal, enabling longitudinal tracking of activity behaviors.

We summarized daily activity metrics by averaging activity patterns over each participant’s monitoring period to derive a representative activity profile. This approach facilitated characterization of individual variability and investigation of associations between activity behaviors and health outcomes. Additionally, these metrics enabled differentiation of activity intensities, offering insights into both total activity volume and its distribution throughout the day.

### Preprocessing

The dataset was accessed via Google BigQuery, extracting all participants with available Fitbit data. SQL queries were used to extract activity summary records, including activity calories, BMR, total calories burned, elevation gain, activity minutes (light, moderate, and vigorous), and total steps.

A cohort filtering process was applied to ensure participants met key inclusion criteria (supplement Figure. 1). First, participants aged 18 years or older were included. Age was determined by comparing the first recorded Fitbit activity date with the participant’s date of birth, retaining only those classified as adults at their first activity record. Next, participants were required to have at least six months (≥ 180 days) of recorded Fitbit activity. This criterion, commonly applied in prior research^34–36^, ensured sufficient data quality and excluded participants with limited activity records that could yield unreliable results. Lastly, only participants with linked EHR data were included. This criterion ensured the availability of EHR data for all participants, allowing assessment of associations between Fitbit-derived activity metrics and clinical outcomes.

### Outcomes and covariates

Study outcomes were derived from diagnostic events recorded in participants’ EHRs. Diagnostic events were identified using International Classification of Diseases (ICD) codes from both ICD-9 and ICD-10 versions. ICD codes were mapped to 1,765 distinct phecodes using the PheWAS catalog’s Phecode Maps^37–39^, ensuring consistent diagnosis categorization. Each phecode was associated with its corresponding phenotype and diagnostic category. To ensure statistical robustness, phecodes with fewer than 100 cases were excluded. Covariates included age and sex, extracted from demographic data.

### Statistical analyses

We used multiple logistic regression models to assess the association between physical activity metrics (e.g., steps, activity calories, and minutes at different activity intensities) and health outcomes. Study outcomes were defined as the presence or absence of specific diagnoses, represented by phecodes derived from EHR data. To enhance robustness, participants diagnosed with a given phecode within the first 180 days of Fitbit monitoring were excluded. Analyses were adjusted for age, sex, and body mass index (BMI) at baseline. Bonferroni correction was applied to adjust for multiple comparisons.

We further performed Cox proportional hazards regression models to explore the association between physical activity metrics and selected chronic diseases. Chronic diseases were selected based on prior research linking them to physical activity^40–47^. Primary predictor variables were Fitbit-derived activity metrics, including activity calories, elevation, activity minutes at different intensities (e.g., fairly active, lightly active, very active), and step count. Outcomes were defined as EHR-recorded diagnosis events, represented by phecodes. For each phecode, baseline activity metrics were calculated as the average values over the initial 180 days following each participant’s first recorded Fitbit activity. Participants with fewer than six months of recorded activity during the baseline period were excluded to maintain data reliability. To mitigate reverse causation, participants diagnosed before or within the first 180 days of Fitbit activity monitoring were excluded from analysis for that specific phecode. Cox models were fitted with individual activity metrics as predictors, adjusting for age, sex, and BMI. The event variable indicated whether a diagnosis occurred during follow-up, while the duration variable represented the number of days from baseline to diagnosis or the end of follow-up. Daily steps were analyzed in 2,000-step increments to assess the impact of step count increases on disease risk. Elevation gain was analyzed in 100-meter increments to evaluate its effect on disease risk. Durations of different activity intensities (e.g., lightly active, very active) and sedentary time were converted to hours to assess the impact of each additional hour on disease risk.

Statistical analyses were performed using Python (v3.8, https://www.python.org/) on the AoU Researcher Workbench, a secure cloud-based platform. The analyses were conducted by the libraries including Pandas^48^, NumPy^49^, Statsmodels^50^, and Lifelines^51^.

### Sensitivity Analysis using BMI for Obesity classification

To evaluate the robustness of our findings, we conducted a sensitivity analysis by defining obesity based on BMI rather than electronic health record (EHR)-based ICD codes. This alternative Classification aimed to capture cases that might not have been explicitly diagnosed in the EHR but met the clinical criteria for obesity (BMI ≥ 30 kg/m^2^). Height and weight records were extracted from the AoU. Participants were classidied as having BMI-defined obesity if they had at least one recorded BMI ≥ 30 kg/m^2^ at any point. To reduce the risk of reverse causation, we excluded participants who had a BMI ≥ 30 kg/m^2^ before the start of Fitbit monitoring or within the first 180 days of Fitbit activity tracking. We repeated the logistic regression and Cox proportional hazards regression models analyses using BMI-defined obesity as the outcome variable.

## Figures and Tables

**Table 1:** Baseline characteristics of study participants

Variable	Fitbit activity participants
Age	52.00 (38.00, 63.00)
Sex	
Female	15,724 (71.41%)
Male	6,214 (28.22%)
Race	
White	17,060 (77.48%)
Black	1,382 (6.28%)
Asian	743 (3.37%)
Others	1,615 (7.33%)
Unknown	1,219 (5.54%)
Ethnicity	
Hispanic or Latino	1,716 (7.79%)
Non-Hispanic or Latino	19,896 (90.36%)
Unknown	407 (1.85%)
Fitbit monitoring length	2.53 (1.42, 4.32) years
Daily steps	6,166 (3,698, 8,600)
Daily elevation	33.92 (4.30, 91.00) meters
Activity intensity (minutes)	
Lightly active	177.02 (118.28, 227.45)
Very active	12.96 (6.03, 24.74)
Fairly active	9.61 (2.62, 18.97)
Sedentary	737.98 (12.05, 931.29)
Calories	
Daily activity calories ^a^	765.68 (484.26, 1,123.62)
Daily BMR calories ^b^	1,474.02 (1,287.86, 1,686.94)
Daily calories burned ^c^	2,101.64 (1,787.42, 2,507.41)

a The number of calories burned for the day during periods the user was active above the sedentary level. This includes both activities burned calories and BMR.

b Total BMR calories burned for the day.

c Total calories burned for the day

**Table 2:** Cox proportional hazards models for the association between Fitbit activity metric and chronic diseases.

	Steps ^a^	Elevation ^b^	Sedentary ^c^	Lightly active ^c^	Very active ^c^
**Chronic disease**	HR (95% CI)				
**Obesity**	**0.846 (0.800–0.895)**	**0.858 (0.777–0.948)**	**1.077 (1.055–1.099)**	**0.848 (0.793–0.907)**	0.712 (0.498–1.018)
**Morbid obesity**	**0.792 (0.731–0.857)**	**0.785 (0.668–0.923)**	**1.054 (1.025–1.083)**	**0.868 (0.793–0.950)**	**0.450 (0.244–0.830)**
**Type 2 diabetes**	**0.776 (0.716–0.843)**	**0.653 (0.531–0.804)**	**1.039 (1.010–1.070)**	**0.821 (0.744–0.906)**	**0.357 (0.200–0.637)**
**Major depressive disorder**	**0.832 (0.770–0.898)**	**0.820 (0.708–0.950)**	1.026 (0.998–1.055)	**0.838 (0.765–0.918)**	**0.565 (0.342–0.935)**
**Obstructive sleep apnea**	**0.835 (0.788–0.885)**	**0.830 (0.746–0.923)**	**1.033 (1.011–1.056)**	**0.873 (0.812–0.939)**	**0.474 (0.322–0.698)**
**Other dyspnea**	**0.841 (0.799–0.886)**	**0.866 (0.793–0.945)**	**1.041 (1.022–1.062)**	**0.909 (0.852–0.970)**	**0.320 (0.221–0.465)**
**Essential hypertension**	**0.875 (0.835–0.917)**	**0.924 (0.862–0.991)**	**1.054 (1.035–1.073)**	**0.883 (0.831–0.934)**	**0.668 (0.507–0.881)**
**Chronic pain syndrome**	0.931 (0.799–1.085)	0.889 (0.683–1.158)	1.051 (0.989–1.116)	**0.778 (0.640–0.946)**	**0.185 (0.044–0.772)**

a. 2,000 steps

b. 100 m

c. Hours

## Data Availability

This study used data from the All of Us Research Program’s Control Tier Dataset v8, available to authorized users on the Researcher Workbench, which can be accessed via https://workbench.researchallofus.org/.
